# Exploring the role of competition induced by non-vaccine serotypes for herd protection following pneumococcal vaccination

**DOI:** 10.1098/rsif.2017.0620

**Published:** 2017-11-01

**Authors:** G. L. Masala, M. Lipsitch, C. Bottomley, S. Flasche

**Affiliations:** 1 Centre for Mathematical Modelling and Infectious Diseases, Department of Infectious disease Epidemiology, London School of Hygiene and Tropical Medicine, Keppel Street, London WC1E 7HT, UK; 2 School of Computing, Electronics and Mathematics, University of Plymouth, Plymouth, UK; 3 Center for Communicable Disease Dynamics, Department of Epidemiology, Harvard T. H. Chan School of Public Health, Boston, Massachusetts

**Keywords:** pneumococcus, vaccination, serotype competition, herd protection

## Abstract

The competitive pressure from non-vaccine serotypes may have helped pneumococcal conjugate vaccines (PCVs) to limit vaccine-type (VT) serotype prevalence. We aimed to investigate if, consequently, the indirect protection of vaccines targeting most pneumococcal serotypes could fall short of the profound effects of current formulations. We compared three previously described pneumococcal models harmonized to simulate 20 serotypes with a combined pre-vaccination prevalence in children younger than 5-years-old of 40%. We simulated vaccines of increasing valency by adding serotypes in order of their competitiveness and explored their ability to reduce VT carriage by 95% within 10 years after introduction. All models predicted that additional valency will reduce indirect vaccine effects and hence the overall vaccine impact on carriage both in children and adults. Consequently, the minimal effective coverage (efficacy against carriage×vaccine coverage) needed to eliminate VT carriage increased with increasing valency. One model predicted this effect to be modest, while the other two predicted that high-valency vaccines may struggle to eliminate VT pneumococci unless vaccine efficacy against carriage can be substantially improved. Similar results were obtained when settings of higher transmission intensity and different PCV formulations were explored. Failure to eliminate carriage as a result of increased valency could lead to overall decreased impact of vaccination if the disease burden caused by the added serotypes is low. Hence, a comparison of vaccine formulations of varying valency, and pan-valent formulations in particular, should consider the invasiveness of targeted serotypes, as well as efficacy against carriage.

## 1. Background

*Streptococcus pneumoniae* is a bacterium that commonly colonizes the nasopharynx, in particular among children. There are over 90 pneumococcal serotypes that differ in their polysaccharide capsule. The capsule has been found to be the major determinant of a serotype's epidemiology as it effects the host's ability to clear the pneumococcus and the probability that colonization leads to pneumococcal disease [1,2]. In comparison to colonization, pneumococcal disease is rare but can include otitis media, pneumonia, bacteraemia or meningitis and aggregates to a major public health burden [3]. In 2000, the first pneumococcal conjugate vaccine (PCV), which provided protection against seven of the most pathogenic pneumococcal capsular serotypes, was licensed and recommended for immunization of infants in the USA [4]. Subsequently, 10- and 13-valent formulations have been licensed and are now being used to prevent pneumococcal disease in more than 130 countries worldwide [5–14]. The incidence of carriage and disease associated with vaccine-type (VT) serotypes declined in vaccinated children, and also in unvaccinated children and adults, after PCVs were introduced into national immunization programmes [15]. However, the overall prevalence of pneumococcal carriage remained approximately constant as non-vaccine-type (NVT) serotypes, i.e. serotypes not targeted by the vaccine, filled the ecological niche [16]. The increased disease from these serotypes has partially offset the benefit of pneumococcal vaccination [15,17,18]. As a result, pneumococcal vaccines that target more or all serotypes are being developed [19].

Previous work has suggested that the competition between VT and NVT serotypes plays an important role in the herd protection observed in the post-PCV era [20–22]. In particular, by reducing acquisition of VT carriage, PCVs give NVTs a competitive advantage over VTs in the nasopharynx. Thus, in vaccinated populations, the presence of NVT in vaccinated hosts provides additional competitive pressure, which combines with the immune protection afforded by the vaccine, to suppress VT colonization. Moreover, at the population level, there is competition between VT and NVT in non-vaccinated hosts, and also the spread of VT is likely inhibited by competition from NVT in non-vaccinated people. In each of these cases, the mechanisms of competition might include direct competition in the nasopharynx [23], induction of innate immune effectors by NVT that also inhibit VT [24] and induction of forms of acquired immunity that also inhibit VT, such as Th17-based and antibody-based immunity to conserved antigens [25–27]. For high-valency pneumococcal vaccines, including those that target proteins common to most pneumococci [19], this hypothesis implies that levels of indirect protection in unvaccinated individuals could fall short of the profound effects that have been observed with the routine use of conjugate vaccines. This would result from effectively losing the benefit of NVT competition and applies even to vaccines with similar vaccine efficacy against pneumococcal carriage and disease.

In this paper, we explore three previously developed dynamic modelling approaches for pneumococcal ecology as to whether they predict a similar qualitative contribution of NVT competition to the indirect effects of pneumococcal vaccination. We harmonized key model parameters that govern vaccine efficacy and pneumococcal epidemiology in the absence of vaccination and explored various vaccine scenarios to better understand the role of competition in providing protection.

## 2. Material and methods

### 2.1. Models

The model by Bottomley *et al.* [21] (M_B) is a deterministic model that represents the pre-PCV steady state of pneumococcal infections in the Gambia. It was used to predict the impact of introduction of PCV13 into the childhood vaccination programme and is fitted to local longitudinal pre-vaccination carriage data. Serotypes are grouped into low, medium or high transmissibility (the probability of onward transmission, given a potentially infectious contact) and high, medium or low clearance rate (the reciprocal of the duration of carriage). Serotypes within the same group share the same properties. Pneumococcal carriers are assumed to gain partial immunity against acquisition of new serotypes during the episode of carriage, which represents the mechanism of serotype competition. A proportion of clearances leads to lifelong immunity against the cleared serotype, which balances competitive advantages to sustain serotype diversity. The default model parametrization is the same as reported in the original manuscript.

The model by Cobey & Lipsitch [28] (M_CL) is an individual-based model that represents a generic high-income setting. Serotypes differed by their intrinsic duration of carriage and their *in vivo* competitive ability. Pneumococcal carriers are assumed to gain partial protection, quantified by the competitive ability of the resident type, against acquisition of additional serotypes during the episode of carriage. This represents the mechanism of competition. Following clearance, the host's susceptibility to any subsequent homologous acquisition as well as the duration of any subsequent carriage episode is reduced. The strength of acquired immunity that reduces carriage duration was fitted to observations from a longitudinal carriage study in infants [29]. Serotype-specific immunity accentuates within-serotype competition, thus providing balancing selection on serotypes, and acquired immunity independent of capsule reduces fitness differences. The simulations presented in this work rely on the default model parametrization. This includes homogeneous mixing, the default rate of acquisition of capsular immunity (*σ* = 0.3) and the default rate of acquisition of non-specific immunity assuming nonlinear reduction in carriage duration (*ε* = 0.25).

The model by Flasche *et al.* [20] (M_F) is an individual-based model that generalizes the most commonly used deterministic pneumococcal model [22,30,31]. It represents a generic high-income country setting. Serotypes differ by their intrinsic duration of carriage. Duration of carriage and susceptibility to acquisition decline with age but are exposure-independent. The acquisition of a pneumococcus triggers both a transient homologous immune response, which represents serotype competition, and a transient heterologous immune response, which represents the mechanism to ensure serotype coexistence. Both immune responses are assumed to prevent additional acquisition of respective serotypes. Unless mentioned otherwise, the simulations presented in this work rely on the default model parametrization, i.e. the duration of specific and non-specific immunity was nine and 18 weeks, respectively.

While there was no formal model selection process, we included dynamic models that simulate serotype competition, that have been used to assess vaccine impact and that span most of the range of alternative assumptions on serotype competition and natural immunity. In particular, M_F is a generalization of the most commonly used compartmental modelling approaches and therefore those approaches were not included [30–38]. An overview of the different modelling approaches in this study is shown in table 1. The main differences between models, in regard to this work, are the assumptions on acquired immunity and the resulting mechanisms for competition and serotype coexistence.

**Table 1.** Summary of the main features of each model of pneumococcal transmission.

	Bottomley *et al.* [21]	Cobey & Lipsitch [28]	Flasche *et al.* [17,20,37,39]
model type	compartmental	individual-based	individual-based
demographics	Gambia	high-income country	high-income country
mixing patterns	homogeneous	homogeneous	age-assortative
natural immunity as a result of infection	*non-specific:* transient immunity for the duration of infection *anticapsular:* chance to develop permanent homologous immunity	*non-specific:* permanent increase in clearance rate, transient reduction in acquisition rate for the duration of infection *anticapsular:* permanent reduction in susceptibility to homologous infection	*non-specific:* transient immunity to heterologous infection *anticapsular:* additional transient immunity to homologous infection *other*: exposure-independent reduction of susceptibility and carriage duration with age
vaccine-induced immunity	like anticapsular natural immunity but higher chance for protection	like anticapsular natural immunity but stronger protection	like anticapsular natural immunity but longer protection

### 2.2. Analyses

We harmonized models to simulate 20 artificial serotypes with a combined pre-vaccination prevalence in children younger than 5 years old of 40%, approximating a high-income setting with moderate transmission, or 70%, approximating a low-income setting with high transmission intensity. In M_B, serotypes were evenly distributed between the three classes, i.e. seven, seven and six serotypes of low, mid and high transmission intensity, respectively. For each model, parameters governing transmission intensity were scaled to achieve the desired prevalence. In M_F, changing the transmission intensity alone was insufficient to achieve the targeted 70% prevalence as the acquired immunity on infection was too strong and long lasting to permit such high levels of carriage prevalence [20]. Hence, once the effects of increasing the transmission intensity saturated, it was kept constant and the duration of specific and non-specific immunity were subsequently decreased to six and 12 weeks, respectively, to achieve the targeted prevalence. As M_B was not age structured, we split all compartments into a less than 5 year old and a 5 years and older compartment retaining all original parameters and constant rate of transition between the strata such that the mean time spent in the less than five stratum was 5 years. To minimize the effects of stochasticity, M_CL was run 10 times and mean values are presented. Stochastic variability on presented outcomes was too small to visualize in the figures so it was omitted. The Simpson index was calculated as a measure of serotype diversity [40].

We explored two vaccines with alternative compositions. Firstly, we compared generic vaccines of increasing valency where serotypes are included in order of their competitiveness. Modelled serotype M1 is the strongest competitor, indicated in all models by the highest prevalence in the pre-vaccine era, and M20 the lowest. Note that the serotype names do not correspond to the numbering conventionally used for pneumococcal serotypes. Secondly, to approximate PCV7, PCV10, PCV13 and PCV15, we modelled inclusion of serotypes into the vaccine in respect of their observed paediatric prevalence rank in global carriage (private communication with Olivier Le Polain 2017). For example, PCV7 targets serotypes 4, 6B, 9 V, 14, 18C, 19F and 23F which are the 18th, 2nd, 6th, 5th, 11th, 1st and 3rd most prevalent serotypes globally. Hence, the PCV7-like vaccine in this work targets model serotypes M1, M2, M3, M5, M6, M11 and M18 (table 2). Serotypes of a lower rank than 20 were not included because the models only consisted of 20 serotypes. We did not consider the 23-valent pneumococcal polysaccharide vaccine as it is not licensed for use in children and does not confer protection against pneumococcal carriage.

**Table 2.** PCV formulations and the ranks of each serotype in terms of its global prevalence according to a review on the global distribution of paediatric pneumococcal carriage.

	serotypes (rank)
PCV7	4 (18), 6B (2), 9 V (6), 14 (5), 18C (11), 19F (1), 23F (3)
PCV10	+1 (31), 5 (38), 7F (32)
PCV13	+3 (9), 6A (4), 19A (7)
PCV15	+22F (27), 33F (24)

M_B and M_F assume that immunity, including vaccine-induced immunity, acts as all-or-nothing immunity (individuals are either immune or not) while the Cobey and Lipsitch model assumes immunity is leaky (individuals gain partial immunity). The models were run to predict the impact of vaccination against each targeted serotype [41]. This assumed 100% vaccine coverage (the proportion of the target population who are immunized) and 55% vaccine efficacy (the reduction in the rate of colonization of a vaccinated compared with an unvaccinated person). Also, the models were run to predict the effective coverage needed to achieve elimination of VT carriage. We defined effective coverage as vaccine efficacy times vaccine coverage. In the two models that assume all-or-nothing vaccine protection, this is equivalent to the fraction of the population that is protected by vaccination, in the M_CL, we assumed 100% population coverage and varying vaccine efficacy. We defined elimination of VT carriage as a reduction in VT carriers of 95% or more. The impact of vaccination is measured as either the percentage reduction in the number of VT carriers or alternatively with any serotype, in year 10 after the start of vaccination if compared to the year before vaccination (steady state). We choose to report only the vaccine impact after 10 years of PCV use as for this work we were only interested in potential qualitative differences in predictions in the mid to long term after vaccine introduction.

## 3. Results

**Figure 1.**
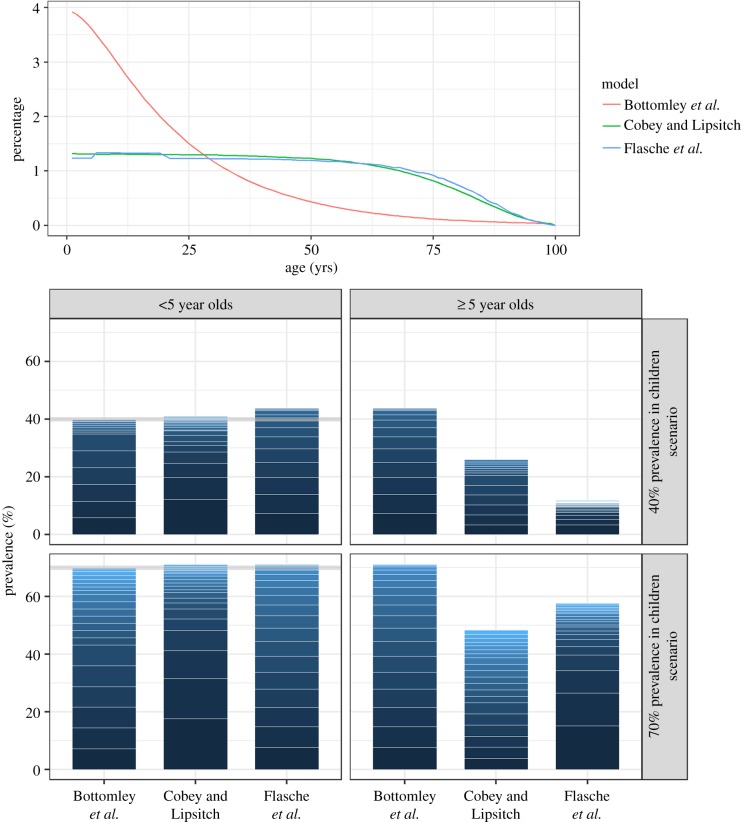
Model demographics and serotypes distribution before the introduction of vaccination. (*a*) The age distribution of the model populations. (*b*) A stacked barplot to illustrate the predicted serotype distributions (stacked prevalence of serotype-specific carriage episodes scaled to the overall carriage prevalence) in children and the rest of the population in low and high transmission settings. The grey line indicates the targeted prevalence. (Online version in colour.)

Models differed in the proportion of children among the simulated population. M_B assumed an age distribution based on the Gambia and hence that children younger than 5 years old make up 20% of the total population. In both other models that represent high-income countries, the corresponding proportion was 5% (figure 1). Pneumococcal carriage prevalence was almost evenly distributed across serotypes in M_F and dominated by fewer serotypes in M_CL. The Simpson indices in children were 0.871, 0.845 and 0.885 in the moderate transmission scenario for M_B, M_CL and M_F and 0.925, 0.862 and 0.927 in the high transmission scenario. In individuals older than 5 years, the predicted prevalence varied between the models (in children less than 5 years, it was fixed at 40% or 70%; figure 1). Carriage prevalence decreases with age except for M_B which was originally designed to be age-independent and hence uses the same parameters for both age groups.

**Figure 2.**
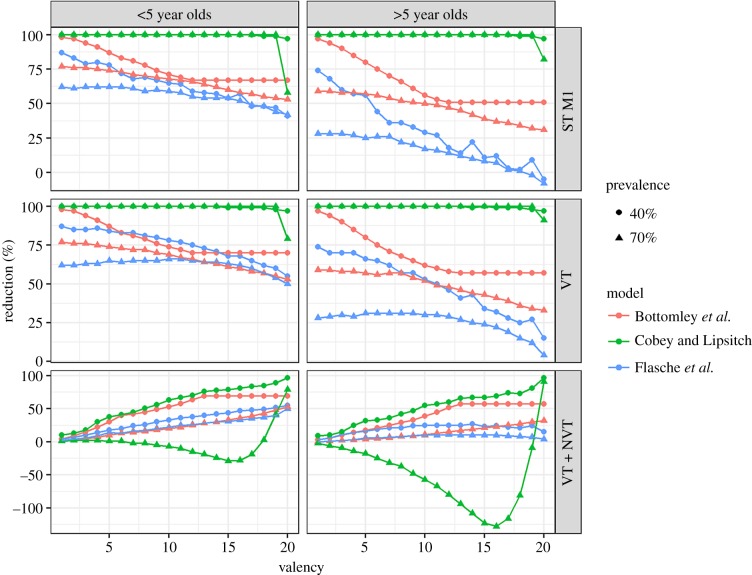
Predicted percentage reduction in the prevalence of pneumococcal carriage (bottom row), VT carriage (middle row) and carriage of the most competitive serotype (top row) 10 years after vaccine introduction, assuming 55% efficacy against acquisition of pneumococcal VTs and 100% coverage. (Online version in colour.)

In the moderate transmission scenario, the vaccine impact against VT carriage 10 years after the start of vaccination of a vaccine with 55% effective coverage decreased with increasing vaccine valency in all three models (figure 2; electronic supplementary material, figure S2). The effect of including more VTs was least visible in M_CL where only inclusion of almost all serotypes (15 or more) reduced the impact on VT carriage to allow VT circulation. The M_B predicted the steepest decrease in vaccine impact on VT prevalence as a result of inclusion of highly and moderately competitive serotypes. However, further inclusion of weakly competitive serotypes, which were hardly carried in this scenario, did not change the impact of vaccination. Similar dynamics were observed for older individuals and the high transmission scenario; however, M_F predicted a small initial increase in vaccine impact on VT carriage before the impact decreased for higher valencies.

The impact of vaccination on all serotype carriage measured 10 years after the start of vaccination with a vaccine with 55% effective coverage generally increased with increasing valency. However, for the high transmission scenario, M_CL predicted an increase in overall pneumococcal carriage through the inclusion of almost all serotypes into the vaccine formulation. Only for valencies of 19 and higher, M_CL predicted a reduction in all serotype carriage prevalence.

**Figure 3.**
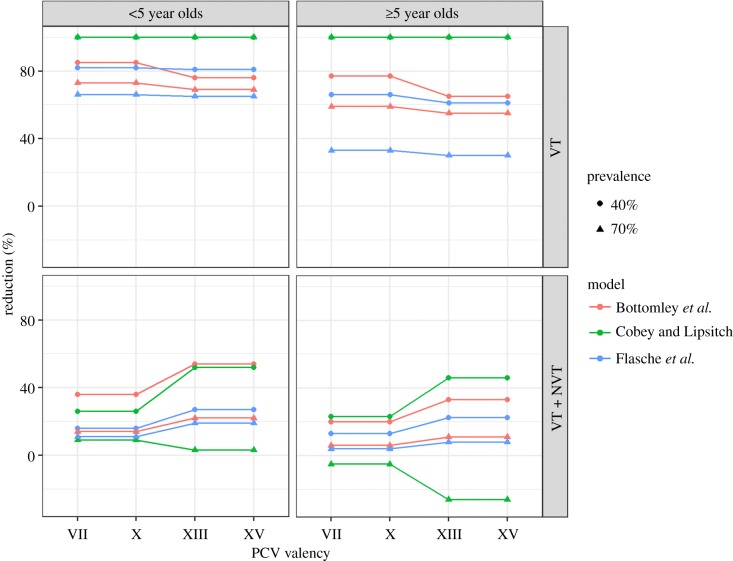
Percentage reduction in the prevalence of pneumococcal carriage 10 years after introduction of a PCV-like vaccine, assuming 55% efficacy against acquisition of pneumococcal VTs and 100% coverage. (Online version in colour.)

The PCV7-like and PCV10-like as well as the PCV13-like and PCV15-like vaccines were indistinguishable. This is because the global prevalence ranks of the respectively added serotypes were larger than the number of serotypes considered in this analysis and hence omitted. All three models predicted that the impact of vaccination against paediatric VT carriage is similar (less than 5% difference) across the PCV-like formulation (figure 3). However, differences in vaccine impact on carriage with any serotype were more pronounced but followed the dynamics of the generic vaccine; i.e. inclusion of more serotypes further reduced carriage prevalence, except in M_CL in the high transmission scenario.

**Figure 4.**
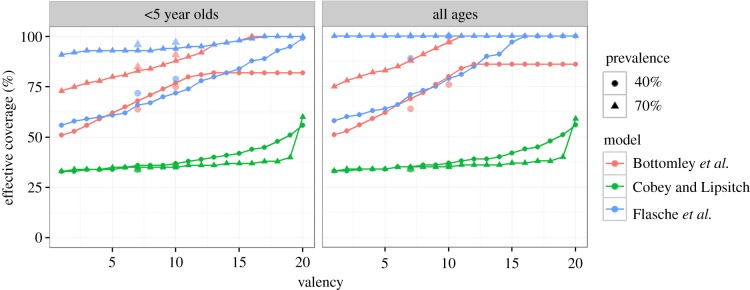
The effective coverage needed to prevent 95% of VT carriage 10 years after the start of vaccination, assuming serotypes are added to the vaccine in order of their carriage prevalence. At valency 7 and 10, the respective effective coverage for PCV7/10-like and PCV13/15-like vaccines are indicated by large dots and triangles in respective colours. (Online version in colour.)

Consistent with the effect of increasing valency on vaccine impact, increasing the valency of the generic vaccine formulation was predicted to increase the effective coverage needed to eliminate VT carriage in both children and older individuals and in both moderate and high transmission intensity settings (figure 4). In all three models VT elimination in the general population required less than 10% additional effective coverage compared with elimination of VT carriage among children. In contrast with the other models, M_CL predicted that addition of up to half of all serotypes into the generic vaccine formulation would not have a profound effect on the effective coverage needed for elimination of VT carriage. Only inclusion of at least 10 serotypes or 15 serotypes in the moderate and high transmission scenario, respectively, would see the need to increase the effective coverage. Elimination of pneumococci using a pan-valent vaccine was impossible in M_F and also for the high transmission scenario in M_B.

For PCV-like vaccines, a similar qualitative behaviour to the generic vaccine formulations was predicted. The three models predicted that the effective coverage needed to eliminate VT carriage in the population increases by 12, 2 and 11%, respectively, for the moderate transmission intensity setting if a PCV13 or 15-like vaccine was used instead of a PCV7 or 10-like vaccine. In the high transmission intensity setting, elimination of VT carriage for the two modelled vaccine formulations was impossible in M_F, required effective coverage larger than 88 and 97% in M_B and required effective coverage of 34 and 37% in M_CL.

## 4. Discussion

PCVs have substantially reduced the burden of pneumococcal disease worldwide. However, through replacement with serotypes not targeted by the vaccines, a sizeable burden remains and has led to ongoing development of vaccines with higher valency. Using qualitative results across three pneumococcal models that span a variety of assumptions on the acquisition of pneumococcal immunity and serotype competition, we here show that serotype competition from NVTs aids VT elimination. Accordingly, we show that targeting an increasing number of serotypes increases the requirements on vaccine efficacy and/or vaccine coverage to achieve elimination of VT carriage. We predict that the relatively small differences in the number of serotypes targeted by current PCV formulations are unlikely to be substantial enough to lead to measurable differences in the ease of VT elimination. However, vaccines that target almost all serotypes may allow continued circulation of the most competitive serotypes, even if the vaccine was given at high coverage and if the vaccine efficacy against carriage was improved over the efficacy of PCVs.

Given the qualitative nature of this comparison, we have only assessed the impact of vaccine valency on the potential of elimination of pneumococcal carriage. The implications of the results for the disease impact of a switch from current PCV formulations to a pan-valent vaccine are complex. Increasing vaccine valency could lead to a net increase in pneumococcal disease burden if two conditions are met: first, the highly competitive serotypes are controlled only through a vaccine with limited valency and are also highly pathogenic and second, the types not targeted by the formulation with limited valency rarely cause disease. In this case, the small disease benefit of controlling more serotypes could be outweighed by the increased circulation of the serotypes in the limited-valency vaccine. The expansion of PCV formulations to incorporate additional serotypes that are responsible for significant amounts of disease has tended to emphasize highly invasive serotypes, thereby minimizing the potential problem we highlight for expansions of the valency of PCVs. Also, a method for selecting serotypes has been proposed that would further minimize the issue of serotype replacement for higher-valency PCVs [42].

In vaccinated persons, these unintended effects might be fully or partially offset through the additional direct vaccine protection against disease, given that PCV formulations thus far have provided greater than 80% protection against disease, with lower efficacy against carriage [41,43–45]. This implies that unintended effects of increased valency of vaccines might be of greatest concern, and thus most deserving of surveillance, in age groups within a population that has not been vaccinated, such as healthy adults. We emphasize that the model-comparison exercise here was designed to assess general trends in the behaviours of the models, rather than to predict specifically how a higher-valency vaccine would act in a particular population. Setting-specific model parametrization is required to allow quantification of the differential impact of vaccine of varying valency on pneumococcal disease.

The principal outcome of this study is that all three models consistently predicted the same qualitative behaviour in response to increasing vaccine valency. We find that serotype competition aids VT elimination and hence that increasing the valency of pneumococcal vaccines increases the herd immunity threshold. For each model, this result was consistent across moderate and high transmission settings and for different vaccine formulations (figures 2 and 3). The finding was also consistent when we explored the impact of a vaccine with a given efficacy and when we estimated the effective coverage needed for VT elimination (figures 2 and 4). Similar to other model-comparison studies that assessed vaccine impact, we did not systematically test the sensitivity of each model's prediction to its ecological assumptions but rather used the differences in assumptions between models to conclude whether our findings are robust [39,46]. In particular, our principal qualitative results were consistent across models despite different model assumptions on the underlying demography, the mechanisms of between serotype competition and the immune memory induced by both pneumococcal infection and vaccination. While none of the three models has been fitted to post-vaccination data, and only M_B was fitted to pre-vaccine data, they all replicate the observed epidemiology in that they predict that a PCV-like vaccine will eliminate most VT carriage within a few years after introduction and that NVTs will almost completely take over the ecological niche so that overall pneumococcal carriage prevalence remains unchanged.

We made the simplifying assumption that serotype-specific direct vaccine effects are the same across vaccines and targeted serotypes and that they follow those of current PCV formulations; i.e. an approximately 55% efficacy against carriage acquisition of any targeted serotype [41]. However, because of the complexities involved in the conjugation procedure of PCVs, it is unlikely that using current techniques, PCVs will be able to target more than 20 of the over 90 pneumococcal serotypes [47]. Vaccines that target common proteins rather than specific capsules, on the other hand, may prevent pneumococcal disease by different mechanisms, e.g. enhanced IL-17A-mediated nasopharyngeal clearance rather than prevention of acquisition [48]. While inference of the differential population impact of specific pneumococcal vaccines would require a more precise parametrization including the focus on a specific setting, the qualitative results of this work are likely to similarly apply.

In a few instances, the models predicted changes to pneumococcal ecology following vaccination that seem counterintuitive at first. In the high transmission scenario, M_CL predicted that overall paediatric carriage prevalence would stay relatively constant (less than 5% change) for vaccines that included up to eight of the most competitive serotypes. However, if more serotypes were targeted by the vaccine, then carriage prevalence increased by up to 40%. Only if at least 19 serotypes were targeted, carriage prevalence was found to decrease (figure 2). This finding is unique to this model because of its inclusion of a gradient in type-specific ability to prevent additional acquisition: carriers of highly competitive serotypes are more protected against acquisition of further serotypes. By protecting against the most competitive serotypes through vaccination, the remaining serotypes are under less pressure from competition to a point where they act almost independently. For vaccines that target between 9 and 18 of all serotypes in the high transmission setting, the prevalence of untargeted serotype in the virtual absence of competition then adds up to exceed the overall prevalence before vaccination (electronic supplementary material, figure S1). Furthermore, in M_CL, the impact of vaccination with low valency vaccines is similar in both transmission settings but slightly higher for moderate to high-valency vaccines in high transmission settings than in moderate transmission settings. By contrast, most models including M_B and M_F predict that transmission intensity and the herd immunity threshold are always positively correlated [49]. In M_CL, that same effect is evident only for vaccines that target all serotypes. For vaccines that target most pneumococci, this model predicts a substantial increase in overall pneumococcal prevalence in high transmission scenarios. The enhanced presence of NVTs in this scenario helps to control VT circulation slightly better than vaccination in a moderate transmission scenario with less transmissible VTs but also lower NVT carriage prevalence and less competition as a result. When assuming 55% effective coverage in the high transmission scenario, M_F predicted that increasing the valency to up to 15 serotypes leads to a small increase in vaccine impact on VT carriage. This is qualitatively different from all other models and scenarios presented here. This is a result of comparing the impact of vaccination on a different number of serotypes. In particular, reduction in carriage prevalence of, for example, serotype ‘1’ as a result of vaccination in the high transmission scenario steadily declines with increasing valency in M_F (figure 2). However, the indirect effect of vaccination against serotypes of lower prevalence is greater and hence comparing the impact of vaccination on all vaccine serotypes includes both the counteracting trends. Only in the high transmission setting, in M_F is a net increase in vaccine effects against VTs predicted for low valency vaccines.

While the models agree well on the qualitative relation between vaccine valency and the herd immunity threshold, we have observed stark differences in the quantitative results. For example, M_CL predicted that much lower effective coverage is required for elimination of VT carriage. Many factors contribute to this observation and some could be addressed by more detailed harmonization of the models to a specific setting. However, two intrinsic model assumptions likely drive this behaviour: (i) the strength of vaccine protection and (ii) the strength of serotype competition. M_CL assumes a relatively strong vaccine protection by reducing susceptibility to VT acquisition permanently after vaccination. M_F assumes vaccine protection, albeit non-leaky, to only hold for 10 years, while M_B also assumes lifelong protection from vaccination, although modelled as all-or-nothing and hence even stronger than in M_CL. While the evidence suggests PCVs to be leaky [50], little is known about upcoming pan-valent vaccines. Vaccine protection has been found to remain present 5 years after completion after childhood immunization [51], but there is some evidence that protection declines over time albeit with a half-life that exceeds 5 years [41]. Further, M_CL has the weakest serotype competition of the models by assuming leaky protection against heterologous acquisition of additional colonizing strains during carriage with the most competitive serotypes providing stronger protection against new acquisition. In comparison, M_B and M_F, respectively, assume competitive exclusion on acquisition and potential co-infection but only after non-leaky heterologous immunity following acquisition has ended. With the relatively weak competition of serotypes in M_CL, a targeted paediatric carriage prevalence is achieved with lower transmission intensity. This in turn will ease elimination of vaccine serotypes in comparison to the other models where transmission is more intense. Recent advances in molecular serotyping methods have shown the pneumococci frequently co-colonize [52,53]. While epidemiological studies suggest that carriage induces both homologous and heterologous protection such protection from a single episode of carriage likely is relatively weak [25,54,55].

## 5. Conclusion

Using three different modelling approaches for pneumococcal ecology that represent a range of alternative assumptions on pneumococcal immunity and serotype competition, we found that NVT competition helps vaccines of limited valencies eliminate VT carriage. This implies that new vaccines that targeted the majority of pneumococcal serotypes will benefit less from NVT competition and are likely to offer less indirect protection than current PCVs. Head-to-head comparison of current PCVs with high-valency vaccines should not only be on the grounds of non-inferiority of direct effects but should also account for indirect effects, and closely monitor IPD endpoints.

## Supplementary Material

Appendix Figure 1:

## Supplementary Material

Appendix Figure 2:

## Supplementary Material

Data

## Data accessibility

All data generated or analysed during this study are included in this article and its electronic supplementary material. The model code is available from the authors on reasonable request.
