# Supplementary figures and images for: Exploring the role of competition induced by non-vaccine serotypes for herd protection following pneumococcal vaccination
Source: J R Soc Interface. 2017 Nov 1;14(136):20170620. doi: 10.1098/rsif.2017.0620 (PMC5721164; doi:10.1098/rsif.2017.0620)

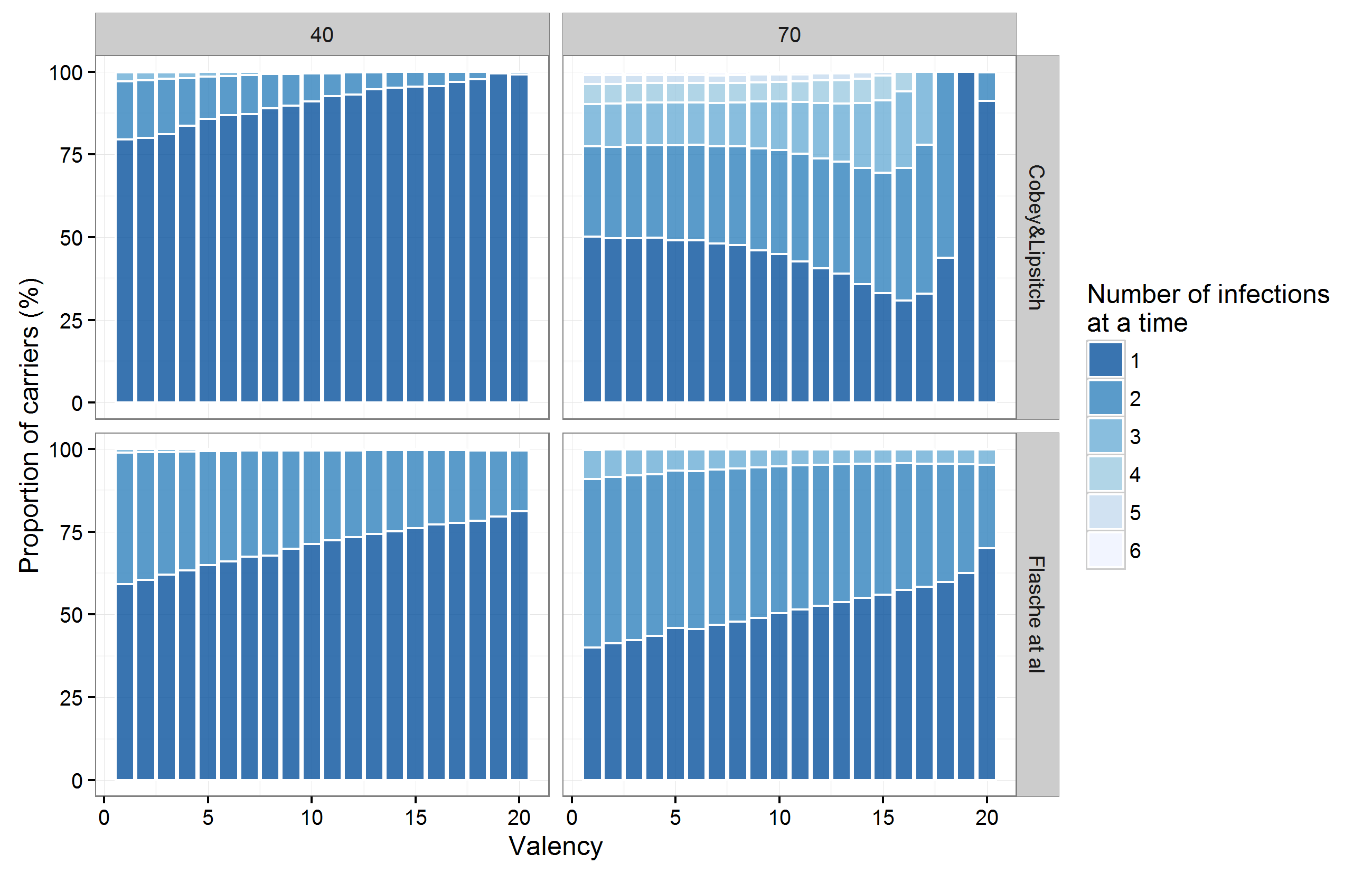

Supplement: Appendix Figure 1: [file rsif20170620supp1.tif]

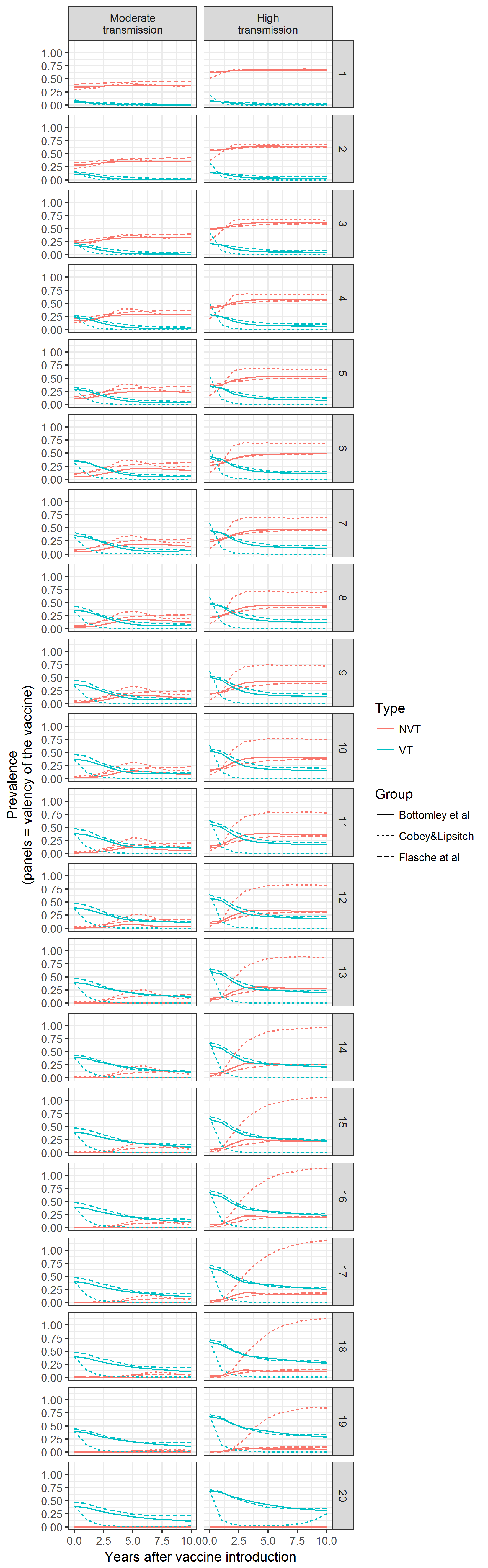

Supplement: Appendix Figure 2: [file rsif20170620supp2.tiff]
